# Idiopathic Nonsurgical Pneumoperitoneum in Healthy Individuals after Endoscopy: Coincidence or Consequence?

**DOI:** 10.1155/2022/7267657

**Published:** 2022-04-05

**Authors:** Jong Soo Lee, Dong-Hoon Yang, Eun Hee Kim, Jin Hwa Park, Sungwon Park, Hye Won Park

**Affiliations:** ^1^Division of Gastroenterology, Health Screening and Promotion Center, Asan Medical Center, University of Ulsan College of Medicine, Seoul, Republic of Korea; ^2^Department of Gastroenterology, Asan Medical Center, University of Ulsan College of Medicine, Seoul, Republic of Korea; ^3^Division of Radiology, Health Screening and Promotion Center, Asan Medical Center, University of Ulsan College of Medicine, Seoul, Republic of Korea

## Abstract

Idiopathic pneumoperitoneum has an unknown etiology despite exploratory laparotomy. However, it may occur without definite abdominal symptoms; thus, adequate management could be in clinical dilemma. We experienced three cases of idiopathic nonsurgical pneumoperitoneum in healthy individuals during a health check-up. Their cases were not accompanied by any relevant etiology or definite abdominal symptoms. All of the three cases exhibited a benign clinical course. The three patients underwent an abdominal computed tomography (CT) scan as part of a health check-up program, which incidentally revealed free air in the right paracolic gutter without evidence of visceral perforation or inflammation. Among the three cases, two patients underwent colonoscopy before abdominal CT, whereas one patient did not. Two cases were completely asymptomatic and were observed without any treatment in the outpatient clinic. Only the third case with minimal symptoms was treated conservatively for a short time. If a small amount of free air typically located in the right paracolic gutter is detected in the absence of perforation during colonoscopy, close observation without unnecessary treatment would be sufficient.

## 1. Introduction

Due to increased health concerns and access to medical imaging, computed tomography (CT) scans are occasionally performed after colonoscopy. On postcolonoscopy CT scans, pneumoperitoneum may occur despite the absence of perforation during the procedure. However, pneumoperitoneum does not always correspond to a perforated viscus that requires surgical intervention. Spontaneous or nonsurgical pneumoperitoneum (NSP) may occur due to a variety of intrathoracic, intra-abdominal, gynecologic, and other miscellaneous etiologies [1]. In particular, idiopathic pneumoperitoneum has an unknown etiology despite exploratory laparotomy [1]. Several cases of idiopathic pneumoperitoneum have been reported previously [2–4], but it is rare in patients without any underlying diseases or relevant symptoms. Moreover, only a few clinicians have provided a management for asymptomatic pneumoperitoneum [3–5]. Hence, adequate management could be a clinical dilemma. We share three cases of idiopathic NSP detected incidentally during a health check-up, which were managed conservatively.

## 2. Case Presentation

### 2.1. Case 1

**Figure 1:**
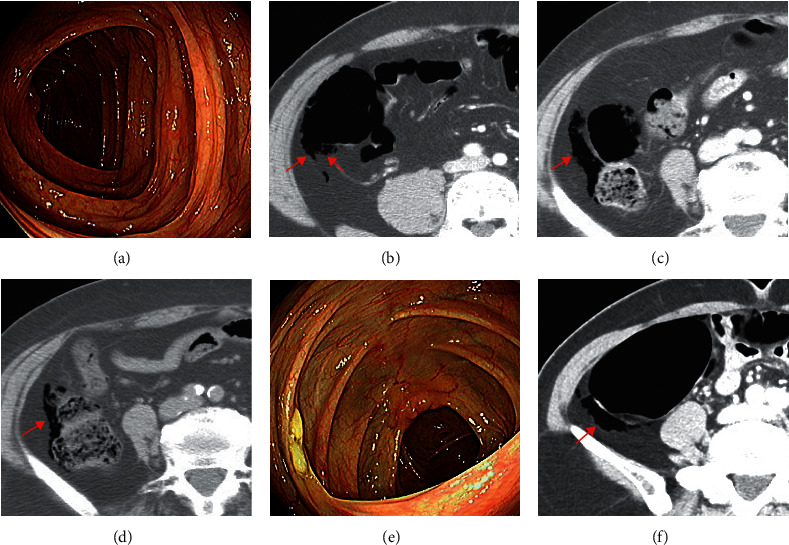
(a) Normal colonoscopic findings in case 1. (b) Abdominal computed tomography (CT) shows a small amount of free air in the right paracolic gutter in case 1. (c) Abdominal CT shows a small amount of free air in the right paracolic gutter in case 2. (d) Follow-up CT scan shows a reduced pneumoperitoneum two months after its initial detection in case 2. (e) Normal colonoscopic findings in case 3. (f) Abdominal CT shows a small amount of free air in the right paracolic gutter in case 3.

A 42-year-old man underwent screening esophagogastroduodenoscopy (EGD) and colonoscopy, which revealed superficial gastritis and normal colonoscopic findings (Figure 1(a)). Room air was used for insufflation. An hour after colonoscopy, he underwent abdominal CT scan as part of a health check-up program, which revealed free air in the right paracolic gutter without any evidence of visceral perforation or inflammation (Figure 1(b)). He did not have any history of abdominal surgery or lung disease. According to the critical value report system in our institution, a radiologist reported the findings to a gastroenterologist and contacted the patient immediately. He was completely asymptomatic postprandial on the day of the test. He was observed without any treatment in the outpatient clinic 10 days later and remained asymptomatic.

### 2.2. Case 2

A 68-year-old woman underwent screening EGD that revealed atrophic gastritis and had a biopsy at the midbody of stomach. Colonoscopy was not performed. After that, abdominal CT was performed as part of a health check-up program. She had a history of total abdominal hysterectomy decades ago and had no lung disease. We contacted the patient on the third day of the exam since free air was recognized on her CT scan (Figure 1(c)). No immediate evaluation or intervention was considered because she did not demonstrate any symptoms. On the follow-up abdominal CT conducted two months later, pneumoperitoneum was slightly decreased (Figure 1(d)). Colonoscopy was performed four months later and showed normal findings.

### 2.3. Case 3

A 66-year-old woman without any medical history underwent screening EGD and colonoscopy (Figure 1(e)), which revealed superficial gastritis and normal colonoscopic findings. After that, abdominal CT was performed as part of a health check-up program. Abdominal CT showed free air in the right paracolic gutter (Figure 1(f)). She was admitted due to minimal postprandial abdominal discomfort, although abdominal examination was not suggestive of peritonitis. She was treated with prophylactic intravenous antibiotics and bowel rest for two days. Follow-up abdominal CT scan two days later showed a slight reduction of intraperitoneal free air. She was discharged on the third day of admission. The patient remained asymptomatic in the outpatient clinic two weeks later.

## 3. Discussion

Pneumoperitoneum usually indicates a perforated viscus, which requires urgent surgical intervention, but the proportion of visceral perforation as the cause of pneumoperitoneum has substantially been reduced [6]. Therefore, clinicians should identify spontaneous pneumoperitoneum or NSP, which can be managed conservatively.

Pneumatosis cystoides intestinalis (PCI) is a frequent cause of NSP [7]. In addition, NSP has been associated with several potential thoracic and gynecological causes and small intestinal diverticulosis [1]. NSP occasionally occurs after colonoscopy. In particular, it has been proposed that microperforation and subsequent air dissection eventually leading to peritoneum via the lymphatic channel are a potential mechanism [1]. In addition, air extravasation through a thinned but intact bowel wall could permit the escape of air without leakage of bowel contents [8]. This can be resolved with conservative management [1, 8]. Finally, idiopathic pneumoperitoneum is a rare clinical entity that has an unclear etiology even after laparotomy [1]. Idiopathic pneumoperitoneum presents with various symptoms including abdominal pain, distension, and elevated inflammatory markers [2]. On the other hand, only a few cases of incidentally detected pneumoperitoneum with thoracic causes, without abdominal symptoms, and with free air, particularly in the subphrenic area, have been reported [3, 4].

In the present cases, we could not identify any clear etiology of pneumoperitoneum radiologically and endoscopically. None of the cases had a history of recent surgery or lung disease or evidence of a perforated viscus, PCI, nor peritonitis. Hence, the present cases were termed as idiopathic NSP.

Previously reported cases of benign pneumoperitoneum after colonoscopy differed from the present ones. In the previous cases, the patients had an underlying condition or definite abdominal symptoms that prompted examination via exploratory laparotomy [5, 9]. In this study, only case 3 exhibited minimal symptoms, for which the patient was subjected to antibiotic treatment and bowel rest for two days. However, it was not clear whether the symptoms were caused by pneumoperitoneum or insufflated air during colonoscopy. Since free air was typically detected in the right paracolic gutter, it could be possible that insufflated air extravasated through the relatively thin right colonic wall. Nevertheless, this mechanism cannot explain case 2, in which the pneumoperitoneum was totally unrelated to colonoscopy. The possibility of ruptured small hidden subserosal PCI cannot be ruled out, considering the frequent location of PCI. Most PCI-associated pneumoperitoneum cases showed typical cystic lesions on colonoscopy or intramural gas of the bowel wall on radiologic exams [10]. These findings were not detected in the present cases. Extraluminal air in NSP usually disappeared within one to two weeks [7]. However, it may last for months as in case 2.

We present three cases of idiopathic NSP with a benign clinical course. In all three cases, CT scans were performed as part of a personalized health check-up program and not to evaluate symptoms after endoscopy. Therefore, this phenomenon is considered as a coincidence rather than a consequence of endoscopy. Once experienced, these conditions would not be difficult to determine. Otherwise, clinical judgement would not be easy. In particular, if a small amount of free air typically located in the right paracolic gutter is detected in the absence of overt perforation during colonoscopy, close observation would be sufficient without unnecessary treatment or bowel rest.
